# Current efforts and challenges facing responses to Monkeypox in United Kingdom

**DOI:** 10.1016/j.bj.2022.07.007

**Published:** 2022-08-05

**Authors:** Ibrahim Idris, Ridwan Olamilekan Adesola

**Affiliations:** a Department of Veterinary Medicine, Faculty of Veterinary Medicine, Usmanu Danfodiyo University, Sokoto, Nigeria; b Department of Veterinary Medicine, Faculty of Veterinary Medicine, University of Ibadan, Ibadan, Oyo State, Nigeria

**Keywords:** Monkeypox virus, United Kingdom, Endemic and surveillance

## Abstract

Despite the extraordinary and exceptional advances in drugs and vaccine development, sophisticated diagnostic facilities in health care settings, strategies in disease prevention and control, emerging and re-emerging infections are still the leading cause of death and suffering amongst human and animal populations with great impact on the world economy. Monkeypox is a viral disease with the potential to spread across the globe through international travel and movements of animals from endemic areas to susceptible populations. Monkeypox virus is an uncommon but endemic viral disease in Africa, but recent outbreaks occurred in Europe, the US, UK, Spain, Portugal, Sweden, and Italy. There is a need to refurbish the healthcare settings and get prepared for future outbreaks, especially in developing countries with poor healthcare delivery services. Scientists and researchers should also focus on developing vaccines, treatment, and preventive measures before the virus spread further.

In May 2022, a cluster of pox-like diseases surfaced Europe, including United State and the United Kingdom. Following laboratory diagnosis, it was confirmed Monkeypox virus disease. Monkeypox is a DNA virus, a member of the genus Orthopoxvirus of the family Poxviridae [1]. The first outbreak of the Monkeypox virus was reported in Denmark, in 1959 from a Monkey in a research center [2], while the first human case was reported by a patient in the Democratic Republic of Congo in the year 1970 [3]. Further outbreaks spread to other countries in Africa including Nigeria, Cameroon, Cote d’Ivoire, Gabon, Liberia, Nigeria, the Republic of the Congo, and Sierra Leone. The first outbreak of the disease outside Africa was reported in May 2003 which is linked to the importation of small animals from Ghana, West Africa, and other cases found in rats and dogs [4]. The disease is associated with fever, headache, malaise, rashes on different areas of the body, and general lymphadenopathy [4].

However, the recent outbreak of Monkeypox virus according to World Health Organization, the first index case of Monkeypox in the United Kingdom was identified on 7th May 2020 from an individual with a travel history to Nigeria, however, a week later 2 new cases were confirmed from Individuals of the same family having no connection with the first case. Four new cases were further identified that also do not have any link with previous cases or any travel history but were men who were identified to have sex with men, and the source of infection is not confirmed.

Therefore, the virus is circulating within the population. Monkeypox is of public health importance due to its morbidity and mortality rate and its economic significance.

This article is aimed at discussing the current effort in controlling and preventing the spread of Monkeypox and the challenges facing its responses in the United Kingdom.

## Epidemiology

Monkeypox virus is an endemic disease in West and Central Africa. However, following its first-ever diagnosis in the Democratic Republic of Congo, precisely in the year 1970, the cases escalate where they appear in many African and European countries. Studies conducted show that the increase in Monkeypox cases among the human population may be attributed to the stoppage of vaccination against smallpox that usually renders a cross-protection against Monkeypox [5]. However, increases in global travel, transportation of animals across countries, and wildlife encroachment are the leading causes increase in the prevalence of the Monkeypox virus, especially in Africa. The natural host reservoir, pathogenesis, and the natural history of the Monkeypox virus are still unclear, thus these are major challenges in understanding the epidemiology of Monkeypox disease [6]. Forested areas have the highest rate of Monkeypox incidence and younger Individuals (low age) who have no history of vaccination against smallpox [7], that's children or younger animals and unvaccinated individuals are at risk of infection. The fashion by which the Monkeypox virus is transmitted from animals to humans is yet to be discovered, but transmission via aerosolized air has been demonstrated in animals, while in humans it's associated with contact with live or dead animals either directly or indirectly [8,9]. The portal of entry of the Monkeypox virus includes mucus membranes such as the nose, mouth, and eyes, respiratory and abrasion.

## Clinical signs of Monkeypox virus infection

The clinical signs of Monkeypox virus manifest 5–21 days (three weeks) following infection, initially it is characterized by severe fever, headache, lymphadenopathy, and muscles pain which is followed by rashes all over the body that appear on different levels from macules, papules, vesicles and pustules [6].

## Prevention and control

Monkeypox virus is a zoonotic disease. Therefore, to prevent the occurrence of this infection there's a need for a multidisciplinary approach (one health approach). Monkeypox virus can be prevented by strict hygiene and sanitation, proper meat inspection, limiting access to hunting wild animals, consumption of bush meat should be avoided at all costs, and proper storage of foodstuffs in households to prevent contamination by rodents.

The spread of the Monkeypox virus can be controlled by the administration of a vaccinia immune globulin [7].

## Treatment

Monkeypox virus infection has no available treatment, however, several antiviral s drugs were employed to treat the infection locally and systemically. The antiviral drugs include Cidofovir, Tecovirimat, and Brincidofovir [10].

## Contemporary efforts

The UK health authorities have established a rapid response team with the mandate of coordinating extensive contact tracing in health care settings and the wider community, that is tracing individuals who encounter infected or confirmed cases.

Moreover, the assessment of individuals that encounters the confirmed cases, is also followed by surveillance for 21 days. Administration of vaccines to individuals with a higher risk of contact. Furthermore, an investigation is also ongoing to determine the route of acquisition that would help in determining other routes of transmission within the population.

## Challenges

The major challenge is that the main source of infection is not identified, which is the major determinant in breaking the chain of disease transmission in a population.

Furthermore, the extent of local transmission is ill-defined, therefore more cases could be identified, or a certain number of individuals may be infected without showing clinical signs of the disease. There would be a low level of herd immunity, as the infection is not endemic in the United Kingdom.

## Conclusion

It is very essential to increase surveillance of the virus in health care settings and within the community, especially in animal populations such as farms, animals, markets, and abattoirs.

People coming from other countries where the disease is endemic should be screened and confirmed free of infection before coming in. Infected individuals should be monitored to prevent the further spread of the virus to susceptible individuals. People should be educated and enlightened on the dangers of bushmeat consumption, zoonosis, the importance of one health, and most importantly implementation of preventive measures and biosecurity against the Monkeypox virus. Finally educating health workers on how to prevent themselves, because they are at higher risk of being infected.

## Conflicts of interest

All the authors declare no conflicts of interest.
